# Identification of Asymptomatic COVID-19 Patients on Chest CT Images Using Transformer-Based or Convolutional Neural Network–Based Deep Learning Models

**DOI:** 10.1007/s10278-022-00754-0

**Published:** 2023-01-03

**Authors:** Minyue Yin, Xiaolong Liang, Zilan Wang, Yijia Zhou, Yu He, Yuhan Xue, Jingwen Gao, Jiaxi Lin, Chenyan Yu, Lu Liu, Xiaolin Liu, Chao Xu, Jinzhou Zhu

**Affiliations:** 1 Department of Gastroenterology, the First Affiliated Hospital of Soochow University, Suzhou, 215006 Jiangsu China; 2 Suzhou Clinical Center of Digestive Diseases, Suzhou, 215006 Jiangsu China; 3 Department of Orthopedics, the First Affiliated Hospital of Soochow University, Suzhou, 215006 Jiangsu China; 4 Department of Neurosurgery, the First Affiliated Hospital of Soochow University, Suzhou, 215006 Jiangsu China; 5 Medical School, Soochow University, Suzhou, 215006 Jiangsu China; 6 Department of Radiotherapy, the First Affiliated Hospital of Soochow University, Suzhou, 215006 Jiangsu China; 7 The 23Rd Ward, Yangzhou Third People’s Hospital, Yangzhou, 225000 Jiangsu China

**Keywords:** Asymptomatic coronavirus-disease-2019 patients, Chest CT images, Convolutional neural networks, Transformer, Deep learning, Transfer learning

## Abstract

Novel coronavirus disease 2019 (COVID-19) has rapidly spread throughout the world; however, it is difficult for clinicians to make early diagnoses. This study is to evaluate the feasibility of using deep learning (DL) models to identify asymptomatic COVID-19 patients based on chest CT images. In this retrospective study, six DL models (Xception, NASNet, ResNet, EfficientNet, ViT, and Swin), based on convolutional neural networks (CNNs) or transformer architectures, were trained to identify asymptomatic patients with COVID-19 on chest CT images. Data from Yangzhou were randomly split into a training set (*n* = 2140) and an internal-validation set (*n* = 360). Data from Suzhou was the external-test set (*n* = 200). Model performance was assessed by the metrics accuracy, recall, and specificity and was compared with the assessments of two radiologists. A total of 2700 chest CT images were collected in this study. In the validation dataset, the Swin model achieved the highest accuracy of 0.994, followed by the EfficientNet model (0.954). The recall and the precision of the Swin model were 0.989 and 1.000, respectively. In the test dataset, the Swin model was still the best and achieved the highest accuracy (0.980). All the DL models performed remarkably better than the two experts. Last, the time on the test set diagnosis spent by two experts—42 min, 17 s (junior); and 29 min, 43 s (senior)—was significantly higher than those of the DL models (all below 2 min). This study evaluated the feasibility of multiple DL models in distinguishing asymptomatic patients with COVID-19 from healthy subjects on chest CT images. It found that a transformer-based model, the Swin model, performed best.

## Background

The novel coronavirus disease 2019 (COVID-19) caused by severe acute respiratory syndrome coronavirus 2 (SARS-CoV-2) has rapidly spread throughout the world, posing a serious danger to global health. Patients with COVID-19 may experience a variety of symptoms, ranging from asymptomatic infection to acute upper respiratory illness, and even severe respiratory failure [1].

Early detection of COVID-19 is aided by a combination of clinical, laboratory, and imaging findings [2]. For example, Ozdemir et al. [3] proposed a novel method for automatic COVID-19 diagnosis using ECG data, with a high accuracy of 96.20%. However, the limited application of ECG tests among patients with COVID-19, as well as variations in the ECG images, makes it less effective and popular than CT examinations. Additionally, Togacar et al. [4] utilized MobileNetV2 and SqueezeNet deep learning (DL) models with SVM classification for differentiating between COVID-19, pneumonia, and normal chest X-ray images. Although they achieved the 99.27% accuracy, the training dataset was greatly limited, and the image preprocessing needed to be normalized and standardized. Furthermore, X-ray cannot visualize the lung lobes at multiple layers, such as with chest computed tomography (CT), which might result in the missed diagnosis. Therefore, chest computed tomography (CT) scans, which are noninvasive and have rapid acquisition speed and excellent sensitivity, are widely used. Previous studies have reported chest CT characteristics in COVID-19–infected individuals, including ground-glass opacities, focal unilateral or bilateral involvement, diffuse and peripheral distribution, and consolidations [5–9]. Furthermore, abnormal chest CT findings compatible with COVID-19 can occur days before detecting SARS-CoV-2 RNA [2, 10]. Thus, the use of chest CT scans, as a rapid supplementary diagnostic measure, may help physicians make a presumptive diagnosis of COVID-19 [2, 11].

Despite its advantages, it is difficult and time-consuming for radiologists to recognize these subtle radiological variations between COVID-19 and pneumonia caused by other etiologies. Due to the enormous number of radiologic tests during the pandemic, this can lead to misdiagnosis, and COVID-19 diagnoses being missed. The usual approach for diagnosing COVID-19 infection is a real-time reverse-transcriptase polymerase chain reaction (RT-PCR) [12]. However, some patients with RT-PCR confirmed SARS-CoV-2 infection may present normal CT features according to radiologists’ interpretations. The RT-PCR was positive, but the lung CT diagnosis was normal, which was referred to be the asymptomatic [12]. This made it difficult for radiologists to detect COVID-19 patients who were asymptomatic [13, 14].

One of the most important duties in reducing the spread of the virus is early detection [12, 15]. However, the increasing number of asymptomatic patients, as well as the possibility of transmission from asymptomatic carriers of SARS-CoV-2, makes it difficult to curtail the spread of this pandemic [16, 17].

Artificial intelligence (AI) has been proved to help in the detection of COVID-19 and to distinguish the difference between COVID-19 and pneumonia caused by other etiologies [18]. To date, various AI-aided diagnostic systems based on X-ray or CT scans have been found to be very promising for diagnosing COVID-19 [18–25]. Harmon et al. [18] proposed a series of DL algorithms for the classification of COVID-19 pneumonia, with high accuracy of 90.8%. Li et al. [19] developed an automated AI system for segmenting and quantifying the COVID-19-infected lung regions on chest CT images, using the UNet structure. Sedik et al. [20] presented two models, based on convolutional neural networks (CNNs) and convolutional long short-term memory, for boosting the accuracy of COVID-19 detection. As we know, however, the research on using AI to help identify the asymptomatic patients is limited. Therefore, this study aims to develop various DL models, based on CNNs or transformer architectures, for detecting asymptomatic COVID-19 patients on chest CT images.

## Materials and Methods

### Chest CT Images

**Fig. 1:**
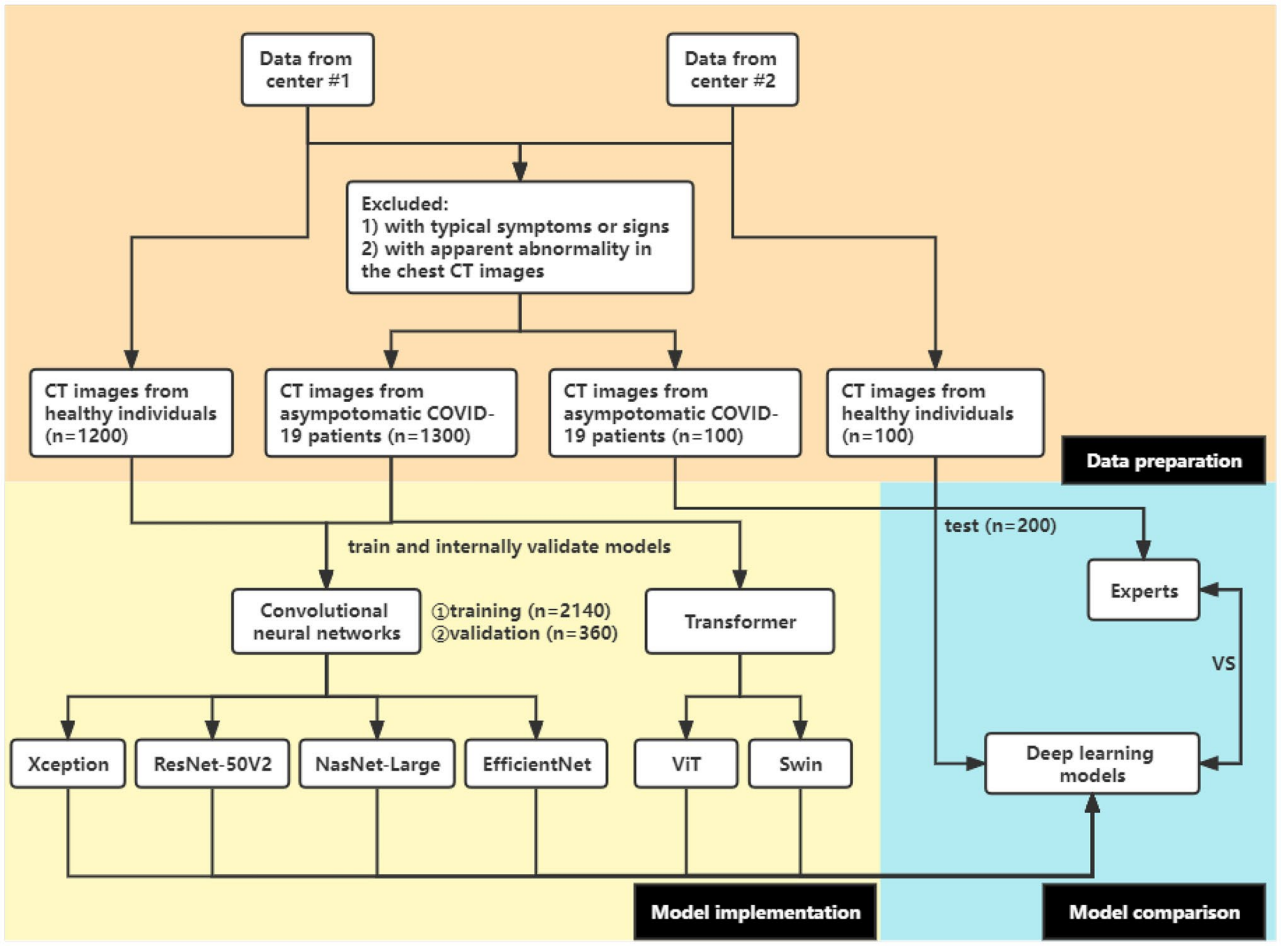
A flowchart of this study

CT images were obtained from Yangzhou Third People’s Hospital (center #1) from August 2020 to June 2021 and from the First Affiliated Hospital of Soochow University (center #2) in 2020; the images were from COVID-19 patients and healthy individuals. All the patients enrolled in this study were confirmed to be positive for COVID-19 by RT-PCR 48 h before or after taking a chest CT exam. The asymptomatic patients were defined as having no evident symptoms. Each CT dataset was reviewed by two radiologists with rich experience, with more than 10 years of chest CT experience in consensus. The images from center #1 (*n* = 2500) were used for training and internally validating models, while the images from center #2 (*n* = 200) were used for externally validating the models. This retrospective study was approved by the ethics and review board of the First Affiliated Hospital of Soochow University. Informed consent was waived. The details are presented in Fig. 1.

### Image Preprocessing

The CT images were downloaded from a medical image cloud platform (www.ftimage.cn). The integrated CT database was saved in PNG format, using a lung window with 5-mm thickness, a 1500 ± 100 Hounsfield unit (HU) window width and a – 600 ± 50 HU window level. All the images were rescaled to 331 × 331 pixels, and then the pixel values were normalized from a range between 0 and 255 to between 0 and 1. To ensure the loss of image information related to pulmonary lesions, a senior radiologist confirmed the PNG format images. No other preprocessing such as image segmentation was carried out.

### Implementation of Pretrained CNN-Based Architectures

We explored four pretrained CNNs, including Xception [26], NASNet-Large [27], ResNet-50V2 [28], and EfficientNet [29], for the classification task. These models were pretrained on the ImageNet database (www.image-net.org). Transfer learning was applied by combining the existing CNN layers with additional activation layers for the learning of the target classification.

#### Input Layer

Each image was normalized as 331 × 331 pixels, padded if necessary and then fed into the pretrained CNN layers.

#### Pretrained CNN Layers

A typical CNN was composed of a convolutional layer, a pooling layer, and a fully connected layer (dense layer), with rectified linear unit (ReLU) activation function. In this study, we selected four CNNs and retained the convolutional layers, each followed by average pooling layers, to extract feature maps from input images by using 2-dimensional filters. Additionally, the ReLU activation function was required to evaluate the output of each layer. All the layers of these pretrained CNN models were frozen.

#### Additional Layers

Subsequent to the pretrained CNN layers, three dense layers with a Sigmoid activation function replaced the original fully connected layers, which acted as a classifier. The output sizes for the pretrained Xception model, the NASNet-Large model, the ResNet-50V2 model, and the EfficientNet model are 2048 × 2048, 4032 × 4032, 2048 × 2048, and 1280 × 1280, respectively. The output sizes for the subsequent three layers are 1024 × 1024, 512 × 512, and 2 × 2.

#### Implementation

We implemented the CNNs based on transfer learning using Keras, where the TensorFlow framework as a backbone provided a Python application programming interface. The Adam optimizer and the binary cross-entropy cost function, with a learning rate of 0.0001 and a batch size of 16, were used to train the models. Firstly, the dataset from center #1 was randomly split into two subsets (85% as a training set, 15% as a validation set) and was fed into the Xception, NASNet-Large, ResNet-50V2, and EfficientNet architectures. Early-stop was applied to determine how many epochs were needed for training a model via callback function for the lowest validation cost. The validation set was used to fine tune the hyperparameters of the models. Lastly, the performance of the models was externally tested by the center #2 dataset.

### Implementation of Transformer-Based Architectures

Compared with the sequential input of CNNs, the transformer architecture is characterized by synchronous input based on the self-attention mechanism. This study selected shifted window transformer (Swin) [30] and vision transformer (ViT) [31] models with the encoder and decoder parts of the transformer. The transformer encoder consists of three main components: input embedding, multihead attention, and feed-forward neural networks.

#### Input Embedding

Due to synchronization, we used sine and cosine functions for position encoding so that two architectures could identify the sequential order. Then, the position encoding matrix was combined with the input images for training the models.

#### Self-Attention Mechanism

Multihead self-attention applied multiple self-attention mechanism to calculate the attention score of the input vectors, which were first separately mapped to query, key, and value vectors. The scaled dot-product attention function was used to calculate scores based on queries, keys, and values, and then a sum of the scores was computed for the self-attention layer’s output. Additionally, a normalized residual connection was added to the output. When training, the learning rates of the two algorithms were both 0.001, and the epochs were both 100.

#### Output Embedding

A feed-forward neural network, followed by layer normalization, was applied to generate feature-embedding vectors. This fully connected network contained a ReLU activation function. The Sigmoid function converted these vector values into predictive probability values.

### Comparison Between Deep Learning Models and Experts

To compare the performance of the models and medical experts, images from the test dataset were identified by two experts, one with 5 years of experience (junior) and the other one with more than 10 years of experiences (senior) in the field of radiology.

### Statistical Analysis

We established DL models using Python software (version: 3.9). We assessed model performance by calculating accuracy, recall, and precision using R software (version: 4.1.0, The R Foundation). More specifically, matrices, consisting of true positives (TP), true negatives (TN), false positives (FP), and false negatives (FN), were enumerated to measure the efficiency of the classifier. Additionally, areas under the receiver operating characteristic curves (AUCs) were calculated to evaluate the robustness of our models. The formulas were as follows: accuracy = (TP + TN)/(TP + FP + FN + TN); precision = TP/(TP + FP); recall = TP/(TP + FN).

## Results

**Fig. 2:**
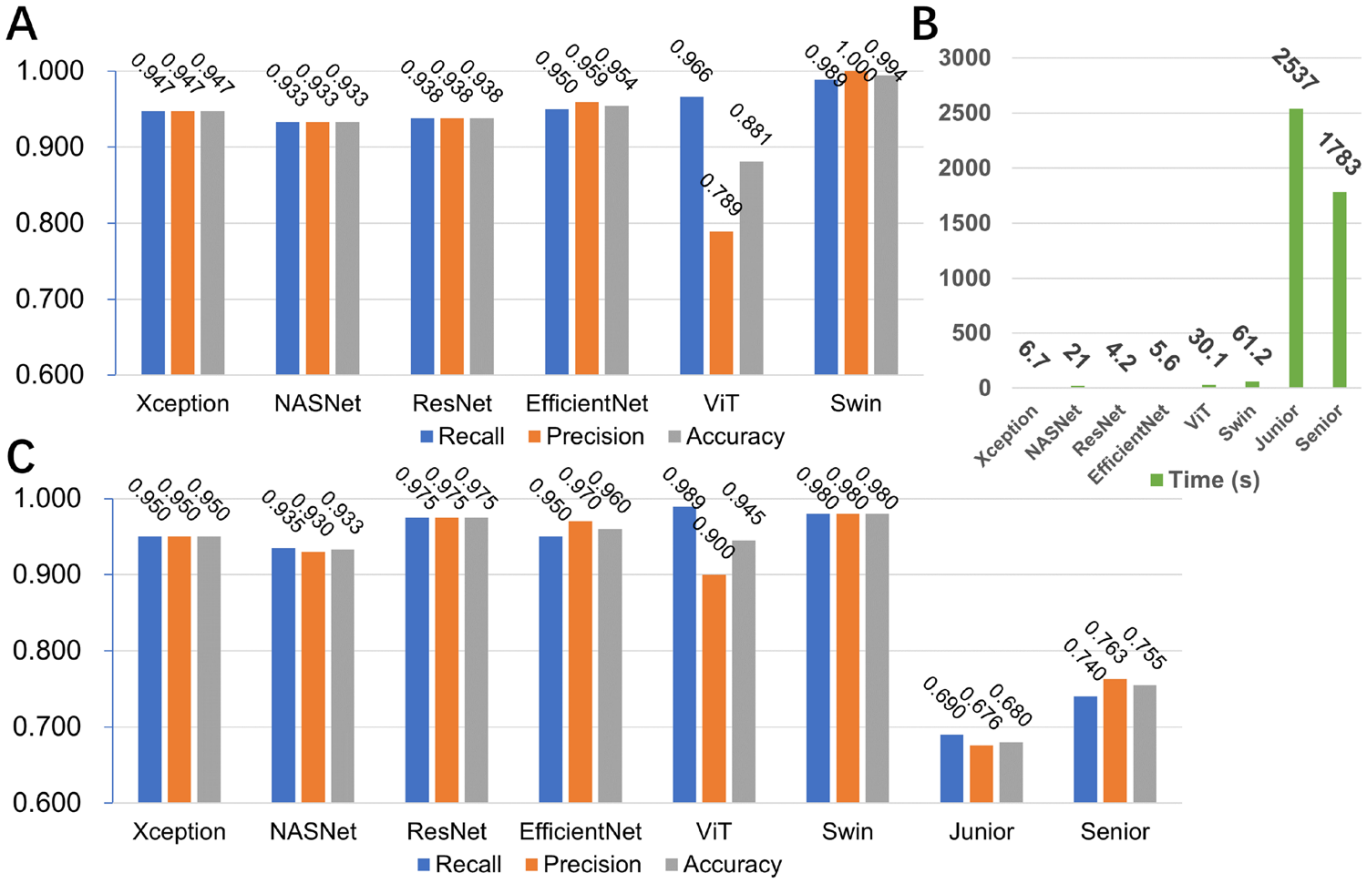
The performance of six models and two experts. **A** The six models’ performance in the validation dataset; **B** the times spent on the test dataset; **C** the six models’ and two experts’ performance in the test dataset

**Fig. 3:**
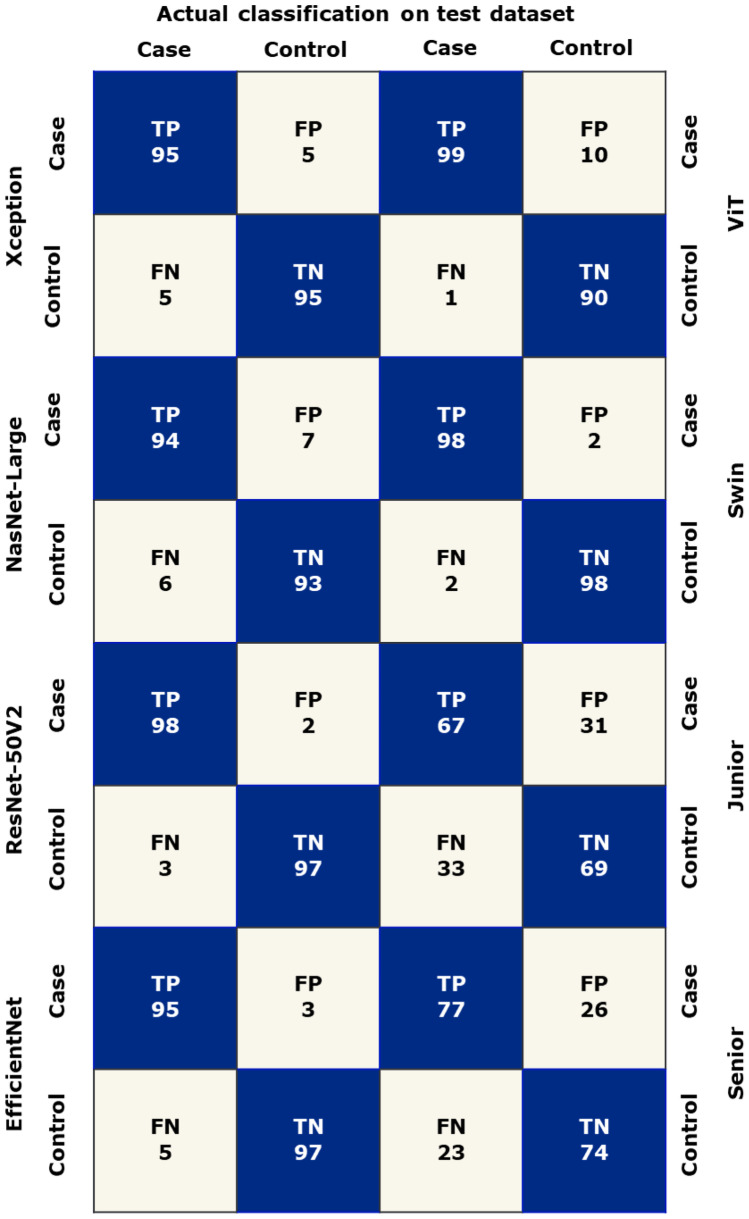
A confusion matrix of two experts and six models. True positives, TP; true negatives, TN; false positives, FP; false negatives, FN

A total of 2700 chest CT images were obtained in this study, consisting of 1400 images of the COVID-19 group and 1300 images of the normal group. Data from center #1 was randomly split into the training set and validation set at a ratio of 8.5:1.5 (2140 vs. 360). The images from center #2 were regarded as the test set (*n* = 200). According to the aforementioned methods, these CT images were fed into the models based on four pretrained CNNs and two transformer architectures for binary classification. The performance of our proposed models is listed in Fig. 2 and shown in Table 1, while the confusion matrix is shown in Fig. 3.

**Table 1:** The performance of six models and two experts

		**Convolutional neural networks**				**Transformer**		**Human**	
		**Xception**	**NasNet-Large**	**ResNet-50V2**	**EfficientNet**	**ViT**	**Swin**	**Junior**	**Senior**
**Training set**	**Recall**	0.969	1.000	0.996	0.984	0.999	0.970	-	-
	**Specificity**	0.967	1.000	0.996	0.991	1.000	0.900	-	-
	**Accuracy**	0.968	1.000	0.996	0.988	1.000	0.935	-	-
	**AUC**	0.995	1.000	1.000	0.999	1.000	0.948	-	-
	**F1-score**	0.968	1.000	0.996	0.988	1.000	0.937	-	-
**Validation set**	**Recall**	0.947	0.933	0.938	0.950	0.972	0.989	-	-
	**Specificity**	0.947	0.933	0.938	0.959	0.789	1.000	-	-
	**Accuracy**	0.947	0.933	0.938	0.954	0.881	0.994	-	-
	**AUC**	0.978	0.968	0.974	0.987	0.881	0.947	-	-
	**F1-score**	0.947	0.933	0.938	0.954	0.891	0.994	-	-
**Test set**	**Recall**	0.950	0.940	0.970	0.950	0.990	0.980	0.690	0.740
	**Specificity**	0.950	0.930	0.980	0.970	0.900	0.980	0.670	0.770
	**Accuracy**	0.950	0.935	0.975	0.960	0.945	0.980	0.680	0.755
	**AUC**	0.987	0.983	0.996	0.991	0.997	0.998	0.680	0.755
	**F1-score**	0.950	0.935	0.975	0.960	0.947	0.980	0.683	0.751
	**Time (s)**	6.7	21	4.2	5.6	30.1	61.2	2537	1783

In the validation dataset, the Swin model achieved the highest accuracy of 0.994, followed by the EfficientNet model (0.954) and Xception (0.947) (Fig. 2A). The recall and the precision of the Swin model were 0.989 and 1.000, respectively.

In regard to the test dataset, the four CNN models were time-friendly and were all less than 10 s. In the two transformer models, it cost 30 s in the ViT model and 61 s in the swin model. However, the two medical experts spent remarkably longer time (1783s by the senior and 2537 s by the junior), as shown in Fig. 2B.

**Fig. 4:**
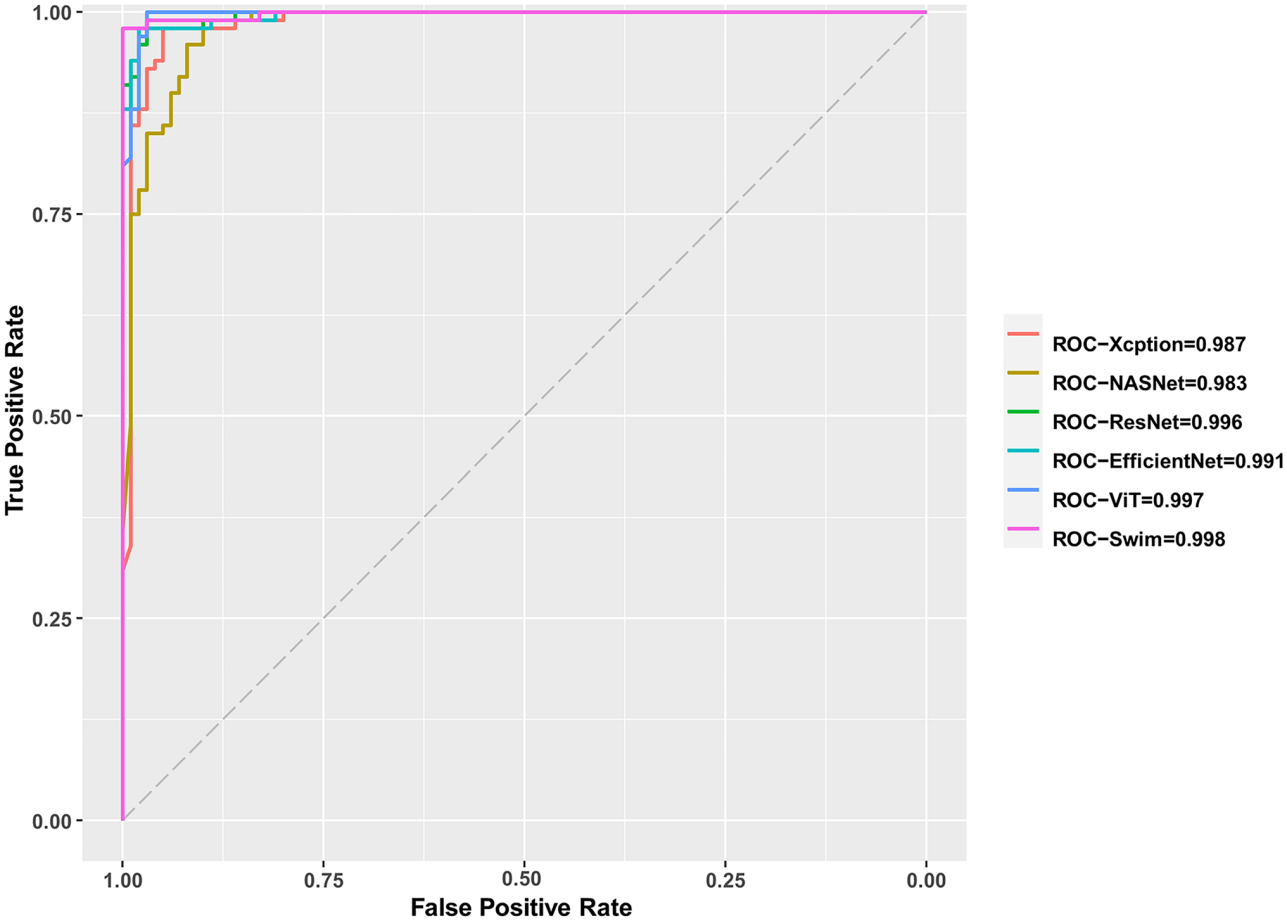
ROC curves of six models with AUCs. ROC, receiver operating characteristic; AUC, area under ROC

In terms of the performance on the test dataset, the swin model was still the best. The accuracy was 0.980, followed by ResNet (0.975) and EfficientNet (0.960). Furthermore, the junior expert presented an accuracy of 0.680, a recall of 0.690, and a precision of 0.676, while the senior expert showed an accuracy of 0.755, a recall of 0.740, and a precision of 0.763, as shown in Fig. 2C. Additionally, the swin model achieved the highest AUC (0.998) among all the models and the two experts, as shown in Table 1 and Fig. 4.

**Fig. 5:**
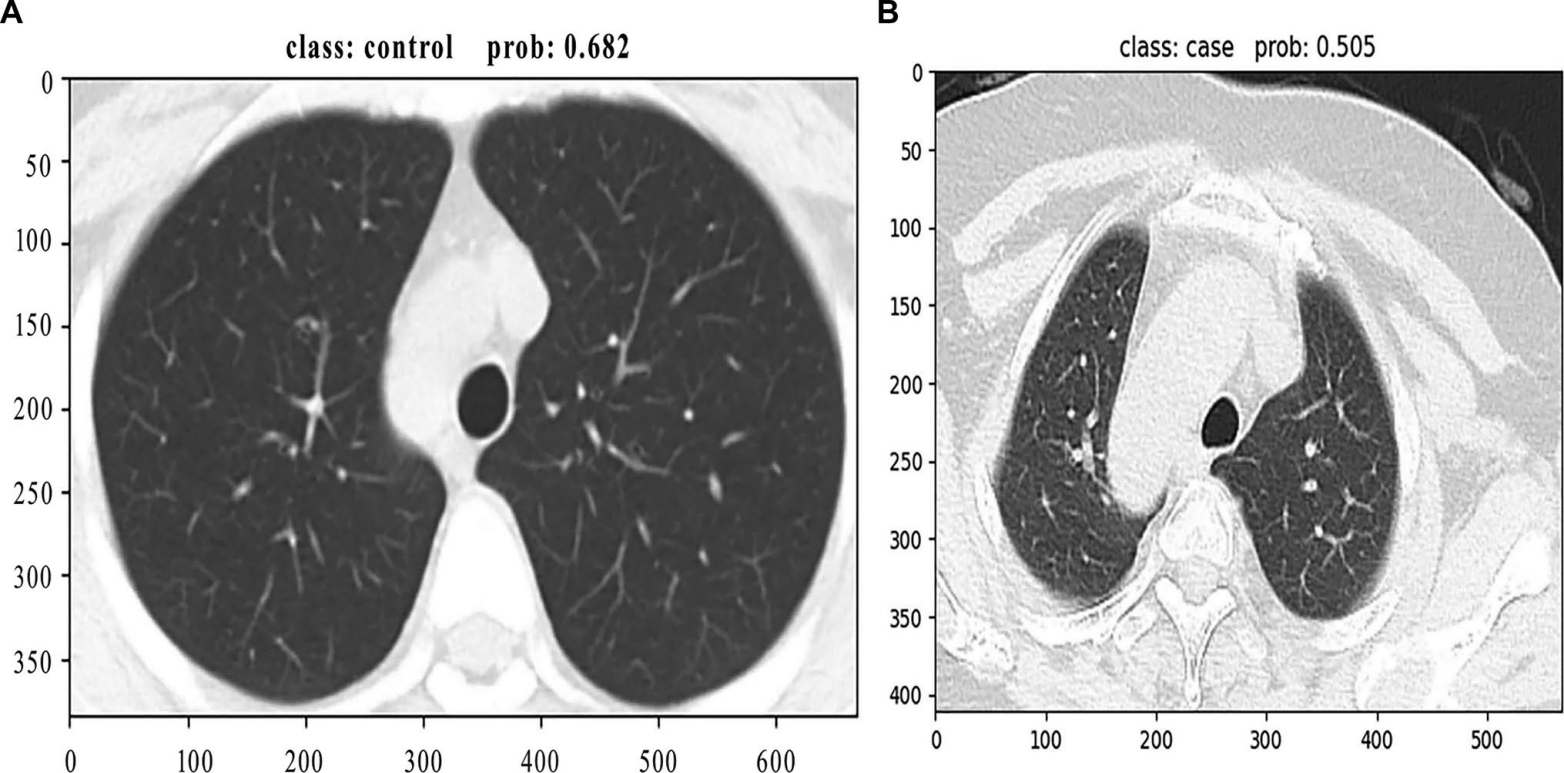
Two misclassified cases predicted by the Swin model. Swin, shifted window transformer. **A** Misdiagnosed as normal image with a probability of 0.682; **B** misdiagnosed as COVID-19 image with a probability of 0.505

Moreover, the two cases misclassified by the swin model are shown in Fig. 5; one was misdiagnosed as a normal image with a probability of 0.682 and another was misdiagnosed as a COVID-19 image with a probability of 0.505.

## Discussion

In this study, we developed four DL models based on CNNs and two transformer architectures to distinguish asymptomatic COVID-19 patients from healthy individuals based on chest CT images. It showed that the transformer-architecture-based model, namely, the swin model, presented the best performance. Furthermore, all the DL models produced better performance than the two experts and spent significantly less time.

Currently, methods for diagnosing COVID-19 are not limited to conventional techniques such as RT-PCR and Chest-CT. Some new modern detection methods have been developed including loop-mediated isothermal amplification (LAMP), droplet digital PCR (dd-PCR), and microarrays for COVID-19 detection [32]. Although these new methods showed high sensitivity and specificity in recent studies, they have not yet been widely used due to the specific environment or the high expenditure [33–35]. Thus, on most occasions, RT-PCR is still the gold standard for diagnosing COVID-19 infection, despite its lack of accuracy and high time consumption [36]. In addition, Chest-CT is also widely adopted clinically as an auxiliary diagnostic method for COVID-19 diagnosis due to its rapid acquisition speed and high sensitivity [8]. However, when compared to RT-PCR tests, Chest-CT has its own advantages, including celerity and convenience. Specifically, it often took at least 6 h for RT-PCR tests to show results, while CT reports could be issued immediately. If COVID-19 spreads widely, large-scale PCR tests will consume more time, human resources, materials, and funds than CT examinations. Furthermore, a chest CT scan can sometimes detect COVID-19 infection based on common CT findings alone even if the RT-PCR test is negative [37–41]. However, in regard to patients who display no clinical symptoms and false-negative RT-PCR results, it may be difficult for radiologists to distinguish between healthy people and asymptomatic infections based on CT features, especially when the newer more modern methods are not accessible. As the number of asymptomatic patients increases, undocumented infections will accelerate the rapid spread of SARS-CoV-2 around the world [16], posing a threat to outbreak control [42].

The past decade has witnessed the emergence of AI as a practicable tool in clinical management [20]. Preliminary studies have confirmed that AI-aided chest CTs had good sensitivities for detecting COVID-19 lung pathologies [43–45]. Sen et al. proposed a model to extract features from chest CT images via CNN and then accurately screened out the most significant COVID-19 characteristics—from the patients’ chest CT images [46]. Bai et al. established an AI model that could differentiate COVID-19 from other pneumonia effectively on chest CTs (accuracy, 96%; and specificity, 96%) and found that AI helped radiologists perform better [47]. In our study, the CT images of asymptomatic patients showed no obvious symptoms, making it difficult to differentiate healthy people from asymptomatic patients. Thus, we adopted four CNN- and two transformer-architecture-based models to evaluate the feasibility of DL in identifying the CT images of asymptomatic COVID-19 patients. On the one hand, we found that the models’ performance was superior to that of the experts, the main reason being that DL algorithms can capture or calculate the subtle differences between images that radiologists cannot detect or understand. DL algorithms are data driven for feature extraction and can automatically obtain deep and specific feature representations based on learning from numerous samples. Moreover, an end-to-end approach allows these algorithms to adapt to the specific medical task requirements [48]. Additionally, DL algorithms can solve with high-dimensional data due to multiple techniques such as the loss function, optimizer, and hidden layers. On the other hand, the results showed that some DL models performed better than others, which we think is because of the different architectures and hyperparameters. CNNs focus on local modeling by convolution kernels, while transformer focuses on global modeling by a self-attention mechanism. Moreover, model accuracy varies with the size of parameters and the throughput. Previous studies suggest that neither CNN nor transformer is completely suitable for all model sizes [49]. Therefore, the model performance of different DL algorithms varies. In addition, these DL models were pretrained on the ImageNet [50], making the process time-saving and less complicated [25], which was easy to use for clinicians.

The novelty of this study is that computer-aided systems are used for processing and analyzing medical images, which greatly improves the value of medical image utilization and the accuracy of clinical diagnosis. Moreover, in the context of AI, the noninvasive diagnosis of CT images of asymptomatic-infected patients provides an important solution to the current challenges to some extent.

However, this study had several limitations. Our data volume was limited, and more datasets from other regions or countries are needed for further verification. Furthermore, these models were trained and tested in retrospective datasets, which might affect the performance in the prospective research. Additionally, the lack of the included patients’ details, such as demographic information, medical history, and even laboratory tests, is another limitation. Multimodal fusion is a potential future trend that could greatly improve model performance, and we will pay more attention to this research direction.

## Conclusions

In conclusion, it was feasible and effective to use DL models for differentiating asymptomatic COVID-19 patients from healthy people on chest CT images. Our study might offer insights into the further application of AI for asymptomatic COVID-19 clinical diagnoses.

## Data Availability

Not applicable. Data are, however, available from the authors upon reasonable request and with permission of the corresponding author.

## Abbreviations

COVID-19: Coronavirus disease 2019
CNNs: Convolutional neural networks
DL: Deep learning
ViT: Vision transformer
Swim: Shifted window transformer
ROC: The receiver operating characteristic
AUCs: Areas under the receiver operating characteristic curve
SARS-CoV-2: Severe acute respiratory syndrome coronavirus 2
RT-PCR: Reverse-transcriptase polymerase chain reaction
CT: Computed tomography
TP: True positives
TF: True negatives
FP: False positives
FN: False negatives
LAMP: Loop-mediated isothermal amplification
RT-LAMP: Reverse transcription-LAMP
dd-PCR: Droplet digital PCR
